# Sequential parametric optimization of methane production from different sources of forest raw material

**DOI:** 10.3389/fmicb.2015.01163

**Published:** 2015-10-20

**Authors:** Leonidas Matsakas, Ulrika Rova, Paul Christakopoulos

**Affiliations:** Biochemical Process Engineering, Division of Chemical Engineering, Department of Civil, Environmental and Natural Resources Engineering, Luleå University of TechnologyLuleå, Sweden

**Keywords:** anaerobic digestion, methane, spruce, pine, birch, hydrothermal pretreatment, enzymatic saccharification, detoxification

## Abstract

The increase in environmental problems and the shortage of fossil fuels have led to the need for action in the development of sustainable and renewable fuels. Methane is produced through anaerobic digestion of organic materials and is a biofuel with very promising characteristics. The success in using methane as a biofuel has resulted in the operation of several commercial-scale plants and the need to exploit novel materials to be used. Forest biomass can serve as an excellent candidate for use as raw material for anaerobic digestion. During this work, both hardwood and softwood species—which are representative of the forests of Sweden—were used for the production of methane. Initially, when untreated forest materials were used for the anaerobic digestion, the yields obtained were very low, even with the addition of enzymes, reaching a maximum of only 40 mL CH_4_/g VS when birch was used. When hydrothermal pretreatment was applied, the enzymatic digestibility improved up to 6.7 times relative to that without pretreatment, and the yield of methane reached up to 254 mL CH_4_/g VS. Then the effect of chemical/enzymatic detoxification was examined, where laccase treatment improved the methane yield from the more harshly pretreated materials while it had no effect on the more mildly pretreated material. Finally, addition of cellulolytic enzymes during the digestion improved the methane yields from spruce and pine, whereas for birch separate saccharification was more beneficial. To achieve high yields in spruce 30 filter paper units (FPU)/g was necessary, whereas 15 FPU/g was enough when pine and birch were used. During this work, the highest methane yields obtained from pine and birch were 179.9 mL CH_4_/g VS and 304.8 mL CH_4_/g VS, respectively. For mildly and severely pretreated spruce, the methane yields reached 259.4 mL CH_4_/g VS and 276.3 mL CH_4_/g VS, respectively. We have shown that forest material can serve as raw material for efficient production of methane. The initially low yields from the untreated materials were significantly improved by the introduction of a hydrothermal pretreatment. Moreover, enzymatic detoxification was beneficial, but mainly for severely pretreated materials. Finally, enzymatic saccharification increased the methane yields even further.

## Introduction

Our economy and production is strongly dependent on the use of fossil fuels, which results in many complications, such as environmental problems and insecurity regarding energy supply. The use of fossil fuels results in the release of huge amounts of carbon dioxide into the atmosphere, which exacerbates global warming. Moreover, apart from carbon dioxide other contaminants such as carbon monoxide, hydrocarbons, particulate matter, etc., are released into the atmosphere with negative effects on public health. One important problem that many countries around the world are facing is the lack of energy security, as most countries need to import fossil fuels. In order to minimize the negative effects of the usage of non-sustainable sources of energy, researchers are focusing on the development of renewable fuels, which can be produced by exploiting domestic sources. The most common sources of biofuels until now have been ethanol, biodiesel, and biogas.

Biogas, a gaseous biofuel, consists mainly of carbon dioxide (CO_2_) and methane (CH_4_), but other minor gases (such as hydrogen sulfide and hydrogen) and moisture are also present. The ratio between the two main gases affects the energy content of the biogas, which has been estimated to be between 18,630 kJ/m^3^ and 26,081 kJ/m^3^ (Romano et al., 2009), whereas natural gas has an energy content of approximately 37,000 kJ/m^3^ (Martin et al., 2013). The main application of biogas is either production of electricity through burning in CHP (combined heat and power) equipment or as a fuel for vehicles (Jeihanipour et al., 2013). In order to be used as a vehicle fuel, the energy content of the biogas must be increased by increasing the concentration of methane. For this reason, carbon dioxide and other gases should be removed by processes such as cryogenic separation, chemical absorption, membrane separation, pressure swing adsorption, and temperature swing adsorption (Kapdi et al., 2005; Molino et al., 2013). Finally, the upgraded biogas is liquefied or compressed. Until recently, only small volumes of biogas have been directed to the transport sector (Naik et al., 2010), although it would be more important to use biogas as a fuel rather than for electricity production (due to the wide range of alternatives).

One of the advantages of using biogas as a fuel is that it can be directly used in vehicles, with only a few modifications required, such as the installation of a special fuel tank. The use of methane as a vehicle fuel contributes to a decrease in the release of greenhouse gases (GHGs), as practically speaking it does not release any GHGs. Moreover, the release of other gases such as carbon monoxide, hydrocarbon, sulfur compounds, and nitrogen oxides is negligible (Swedish Gas Association, 2011). Nevertheless, production of biogas is considered better from a resource efficiency point of view with an output to input ratio reaching values as high as 28 (Jeihanipour et al., 2013; Zheng et al., 2014). For comparison, the same ratio for bioethanol is calculated to be 3.7 for the Brazilian model (where sugarcane is the raw material) and only 1.1 for the U.S. model (where corn is used; De Oliveira et al., 2005).

Biogas production takes place through anaerobic digestion, which is a complex multi-step biochemical process. Each step of this process is catalyzed by a different group of microorganisms, where all of them work together as a ‘community’ to convert organic molecules to biogas (Li et al., 2011; Parawira, 2012). Anaerobic digestion consists of a hydrolysis step, an acidogenesis step, an acetogenesis step, and a methanogenesis step. In the first step, complex molecules such as polysaccharides are hydrolyzed to more simple molecules, which in the second step are converted to volatile fatty acids (VFAs) and alcohols. VFAs longer than acetic acid are converted to acetate, CO_2_, and H2 by the acetogens, whereas in the final step VFAs are converted to CH_4_ and CO_2_ by methanogens (Mshandete et al., 2005; Adu-Gyamfi et al., 2012). Due to the fact that the rate of VFA production is higher than the rate of VFA consumption, if the process is not well balanced there is a risk of accumulation of VFAs—which will result in a decrease in the pH and subsequent inhibition of methanogenesis. Anaerobic digestion is normally performed under mesophilic conditions (25–35°C) or thermophilic conditions (45–60°C) with the thermophilic digestion presenting some positive characteristics such as higher methanogenic activity, a faster process, and fewer contamination problems (Lesteur et al., 2010; Xia et al., 2013). At the end of the anaerobic digestion, the digestate produced can be used as a bio-fertilizer as it is rich in nitrogen, phosphorus, and potassium, which also presents peculiar rheology (Adu-Gyamfi et al., 2012; Kafle et al., 2014).

Currently, the most commonly used materials for biogas production are animal manure, food wastes, and municipal wastewaters. Use of these raw materials has proven to be very beneficial, as high yields have been already been achieved, resulting in the construction and operation of commercial units. On the other hand, the increasing need for biogas results in an urgent need to incorporate novel renewable raw materials in the biogas production line. Lignocellulosic biomass is an excellent candidate for use as a raw material, and has attracted much research interest in recent years. It can be derived from a variety of sources such as agricultural residues and forest residues. Forestry is a very important part of the Swedish economy, and the total standing volume of forests in Sweden is approximately 3,000 million m^3^ with an annual increase in the standing volume of 40 million m^3^ (www.svenskttra.se), which is a result of the very good forest management. The main tree species in Sweden are Norway spruce (*Picea abies*), Scots pine (*Pinus sylvestris*), and birch (*Betula pendula* and *B. pubescens*), which make up 41, 40, and 18% of the total standing volume of forests^1^.

Lignocellulosic materials have low digestibility and the methane yields are therefore low, making a pretreatment step prior to digestion necessary. Different kinds of pretreatments have been evaluated in order to improve the methane yield from forest biomass, such as steam explosion (Nakamura and Mtui, 2003; Horn et al., 2011), ionic liquids (Teghammar et al., 2012; Kabir et al., 2014), organosolv (Kabir et al., 2015), and supercritical water (Yoshida et al., 2010). On the other hand, pretreatment could result in the degradation of sugars and generation of inhibitory compounds that could hinder the anaerobic digestion. In order to reduce the level of inhibitors, a detoxification process could be used. Different detoxification techniques have already been evaluated during ethanol fermentation—such as treatment with reducing agents (Alriksson et al., 2011; Xiros and Olsson, 2014), with laccase (Moreno et al., 2013), or with linear polyethylenimine solutions (Cannela et al., 2014)—but little is known about the effect of detoxification on anaerobic digestion. Finally, despite the fact that the microorganisms that are present in the sludge are capable of exploiting the insoluble carbohydrates, our group has shown previously that addition of hydrolytic enzymes can increase the methane yields (Matsakas et al., 2014).

For this reason, the aim of this work was to evaluate the possibility of using the main tree species in Sweden as raw materials for anaerobic digestion. In addition, the effects of hydrothermal pretreatment, slurry detoxification, and enzymatic hydrolysis on the methane yield were also investigated.

## Materials and Methods

### Raw Materials and Enzymes

Untreated forest residues were provided by SLU (Umeå, Sweden). The total solids (TS) and volatile solids (VS) of the materials were as follows (w/w): spruce, 90.81% TS and 90.49% VS; pine, 91.45% TS and 91.26% VS; birch, 92.07% TS and 91.86% VS. The thermophilic anaerobic sludge used during this work was collected from a biogas plant in Boden, Sweden, where sewage sludge and food waste are co-digested.

The cellulolytic enzymes used during this work were the commercial enzyme solutions Celluclast^®^ 1.5L and Novozym^®^ 188 (Novozymes A/S, Bagsværd, Denmark) at a ratio of 5:1 v/v. The activity of the mixture was measured to be equal to 83 filter paper units (FPU)/mL. The enzymatic detoxification was performed using a laccase from the fungus *Pycnoporus cinnabarinus*, which was kindly provided by Beldem (Belgium) with a declared activity of 13 IU/mL.

### Pretreatment of Forest Residues

Hydrothermal pretreatment took place at the SEKAB plant in Örnsköldsvik (Sweden) in a continuous mode unit. Sulfur dioxide was used as a catalyst during the pretreatment at a concentration of 1 kg per 40 kg of biomass (Soudham et al., 2011). The different source of biomass were pretreated under different combinations of holding time and temperature (**Table 1**) and the pH after the pretreatment varied depending on the severity of the process (**Table 1**). The slurries obtained had a different content of solids, which in terms of TS and VS were as follows (w/w): severe pretreated spruce, 25.81% TS and 25.55% VS; mild pretreated spruce, 23.48% TS and 23.28% VS; pine, 19.51% TS and 19.38% VS; birch, 21.69% TS and 21.60% VS.

**Table 1:** Pretreatment conditions of the materials used during this work.

Raw material		Temperature (°C)	Holding time (min)	pH^∗^	Average SF
Spruce	Severe	212	4–8	1.6–1.8	4.08
	Mild	200	4–8	1.8–2.0	3.72
Pine		210–215	5	1.5–1.7	4.01
Birch		190	4–6	1.8–2.0	3.35

*^∗^The value of pH is for the slurry after the pretreatment.*

### Enzymatic Saccharification of Pretreated Slurries

In order to evaluate the effect of the pretreatment on the enzymatic digestibility of the slurries, a series of enzymatic saccharification experiments was performed. During these experiments, both untreated and treated materials were included. When pretreated materials were used, in order to be easier to handle, low quantities of the slurries were dried at 70°C before the experiments. Enzymatic hydrolysis was performed at a solids content 3% w/v in an eppendorf thermomixer at 50°C for 24 h. The pH of the solution was adjusted to 5 using 100 mM citrate-phosphate buffer. Sodium azide at a concentration of 0.01% w/v was added to the mixture in order to prevent microbial contamination. Samples at 0 and 24 h of incubation were centrifuged and the supernatants were analyzed for soluble sugars.

### Detoxification of the Slurries

Two different detoxification processes were evaluated during this work, one chemical and one enzymatic. The chemical detoxification consisted of treatment with sodium dithionite. The concentration of sodium dithionite was set at 10 mM or 1 mM, and the treatment took place at the same time as the anaerobic digestion. After the addition of the salt, the pH of the sludge was measured in order to ensure that it was not affected.

The treatment with laccase took place prior to digestion, for 12 h at 50°C under aerobic conditions. The slurries were diluted with distilled water in order to obtain a final solids concentration of 10% w/v and the enzyme load used was 10 IU/g VS. Finally, the pH of the slurry was increased to 5.5 by adding appropriate amounts of NaOH.

### Enzymatic Treatment of the Slurries with Cellulolytic Enzymes

Two different processes were used for enzymatic treatment of the slurries, namely treatment along with the digestion and pre-saccharification. During simultaneous treatment, the cellulolytic enzymes were added to the sludge at the start of digestion. On the other hand, when a pre-saccharification step was included, the slurries were diluted to a solids concentration of 10% w/v and the saccharification took place at 50°C for 12 h. The pH was also kept at 5.5 by addition of NaOH. Two enzyme loadings were applied in both configurations (15 FPU/g VS and 30 FPU/g VS), in order to evaluate the effect of the enzyme activity on the methane yields. Finally, it is worth mentioning that when laccase treatment was applied for a specific slurry, then both enzymatic treatments were performed at the same time (in the case of pre-saccharification).

### Biochemical Methane Potential (BMP) Tests

In order to evaluate the digestibility of the substrates, BMP tests were used. More specifically, the BMP tests were performed using the AMPTS II system (Bioprocess Control AB, Lund, Sweden). The system has three parts: the digestion flask, the CO_2_ fixation unit, and the flow meter unit. The digestion flasks are 500-mL glass bottles; these were filled with 400 g of sludge and substrate. The CO2 fixation unit consists of 100-mL glass bottles; these were filled with approximately 80 mL of 3 M NaOH in order to ‘trap’ all the other gases except methane. Thymolphthalein was added as a pH indicator, in order to check that the solution remained active. Finally, the flow meter unit consists of an array of flow meter cells where the methane is counted. The values of methane volume are correlated to normalized volume.

In each batch of experiments, two control samples were also included. The first one contained the sludge, in order to calculate the methane produced by the organic load that remained in the sludge, and the second control contained the enzyme (cellulases or laccase, alone or together), which was used in order to count the amount of methane produced from the digestion of the enzymes. The values of methane from both the sludge and the enzymes were subtracted from the total amount of methane, in order to calculate the methane yield from the substrate alone. Finally, a positive control was also included where avicel cellulose was used as raw material, in order to evaluate the quality of the sludge.

The digestions were performed in duplicate and the I/S ratio (inoculum-to-solid ratio) was set at 2 in terms of VS. The digestion was carried out at 55°C, and each flask was supplemented with mineral and salt solution, the composition of which is described elsewhere (Antonopoulou and Lyberatos, 2013). Prior to digestion, the flasks were sparged for approximately 1.5 min with nitrogen in order to remove the oxygen.

### Analytical Methods

The TS and ash contents were determined gravimetrically after drying for 24 h at 105°C and burning for 2 h at 550°C, respectively. In order to determine the VS content, the ash content was subtracted from the TS content. Total reducing sugars during the enzymatic hydrolysis experiments were determined according to the DNS method (Miller, 1959). The enzymatic activity of the mixture of Celluclast^®^ 1.5L and Novozym^®^ 188 was determined by standard filter paper activity method (Ghose, 1987). The sugars from the structural carbohydrates analysis and also the inhibitors were determined using an HPLC apparatus equipped with a Series 200 RI (refractive index) detector (PerkinElmer). More specifically, during the sugar analysis an Aminex HPX-87P was used with ultra-pure water as mobile phase. The flow rate was set at 6 mL/min and the column was kept at 85°C. For the inhibitors, an Aminex HPX-87H column was used with 5 mM H_2_SO_4_ as mobile phase. The flow rate was set at 0.6 mL/min and the column was kept at 65°C.

## Results

### Methane Yields from Untreated Materials

**FIGURE 1:**
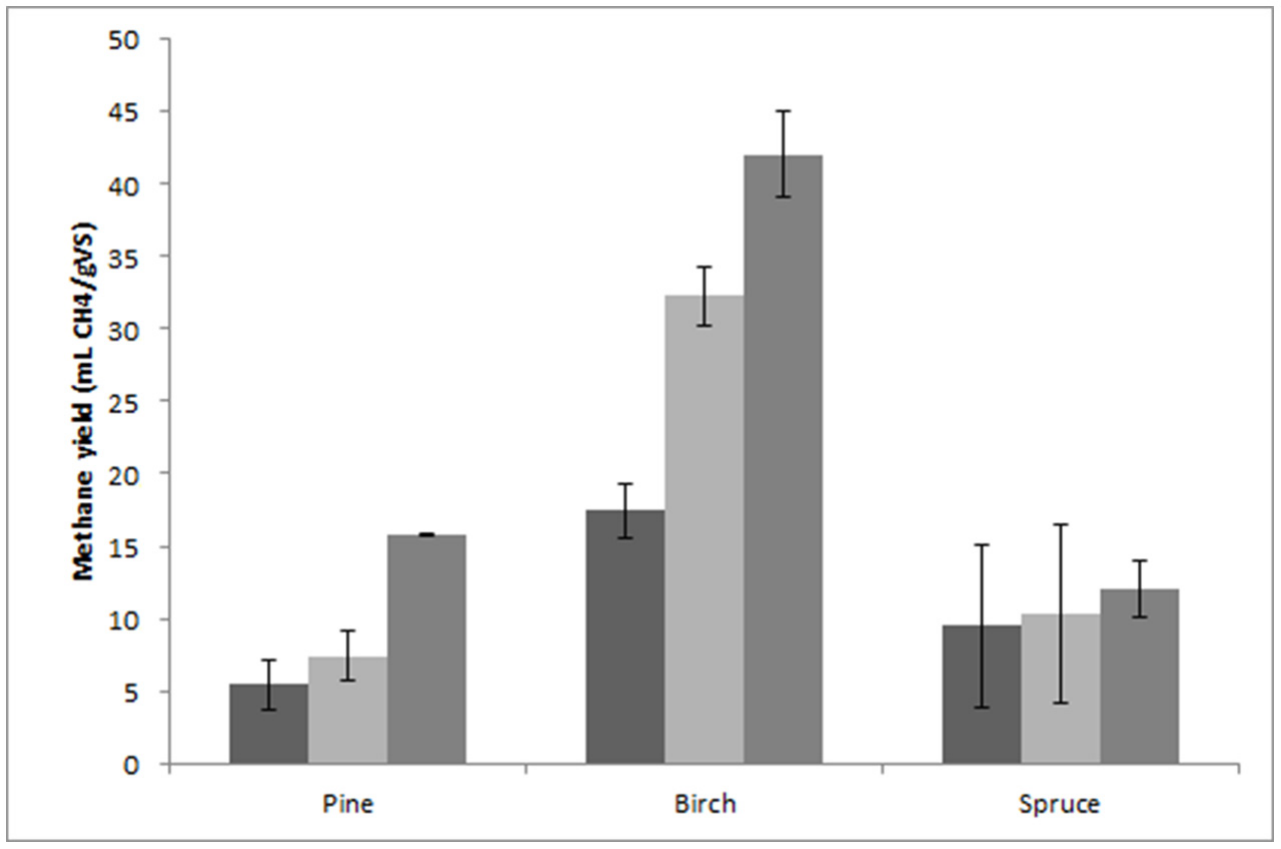
**Methane yield from untreated materials without the addition of enzymes (first bar), and with the addition of 15 FPU/g enzyme (second bar) and 30 FPU/g enzyme (third bar)**.

In the initial stage of this work, the possibility of using untreated spruce, pine, and birch as raw materials for anaerobic digestion was evaluated. The highest yield obtained was observed with birch, and it only reached 17.5 ± 1.9 mL CH_4_/g VS, whereas the yields obtained from spruce and pine were even lower (**Figure 1**). In an attempt to improve the methane yield, we studied the effect of addition of cellulolytic enzymes. As previously described, anaerobic sludge is capable of hydrolyzing insoluble carbohydrates such as cellulose. On the other hand, addition of external enzymes can facilitate this process and improve methane yields. Two different enzyme loadings were applied, namely 15 FPU/g and 30 FPU/g. The presence of the enzymes improved the yield of methane from all the materials, with the highest yield (40 ± 3 mL CH_4_/g VS) being obtained when 30 FPU/g was used for birch (**Figure 1**). On the other hand, addition of enzyme slightly improved the yield obtained from spruce. Despite the fact that the yields were improved compared to digestion without the addition of enzymes, the yields were low to be considered as an efficient anaerobic digestion.

### Effect of Hydrothermal Pretreatment on the Digestibility of Residues and on Methane Yield

During this step of the work, a hydrothermal pretreatment with SO_2_ as a catalyst was used at the SEKAB plant (Örnsköldsvik, Sweden). The materials were treated at different combinations of temperature and holding time, resulting in different pH after pretreatment (**Table 1**). The treated materials were collected as slurry with a total solid content varying from 19.5 to 25.8% w/v, and the composition of the liquid (in inhibitors) and solid fractions is presented in **Table 2**.

**Table 2:** Structural and inhibitor analysis of the solid and liquid fraction, respectively.

Raw material	Solid Fraction (% w/w)				Liquid Fraction (g/L)			
	Glucan	Xylan	Lignin	Ash	Acetic acid	Levulinic acid	HMF	Furfural
Severe	31.96	ND	46.69	0.36	10.24	5.30	6.11	1.87
Mild	47.61	1.06	46.77	0.17	6.39	ND	1.27	1.21
Pine	46.24	0.76	47.49	0.26	5.50	2.28	1.70	1.48
Birch	47.73	1.82	32.47	0.10	19.17	ND	0.37	2.76

*ND, not detected.*

**FIGURE 2:**
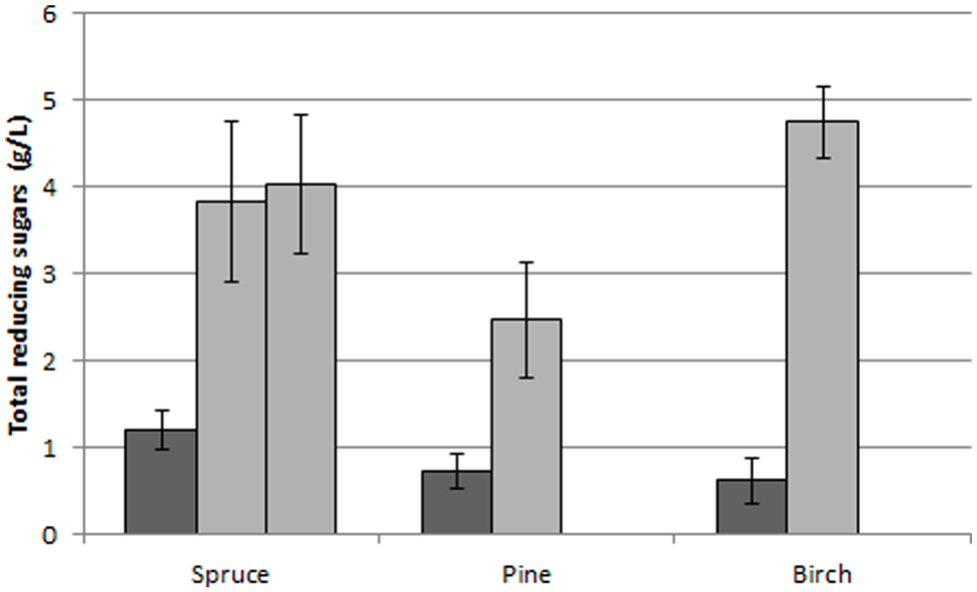
**Release of TRS from untreated materials (dark gray) and pretreated materials (light gray) after 24 h of enzymatic digestion with an enzyme load of 10 FPU/g and solids content of 3% w/w.** For pretreated spruce, the first and the second bar represent mild and severe treatment conditions, respectively.

At a first step, the digestibility of the pretreated materials was examined. For this reason the slurries obtained after the pretreatment were digested at low solids concentration using an enzyme load of 10 FPU/g solids. Pretreatment improved the release of reducing sugars up to 6.7 times compared to the untreated materials (**Figure 2**). The greatest improvement was observed with birch, whereas for spruce severe pretreatment improved only slightly the saccharification compared to the mild pretreatment.

**FIGURE 3:**
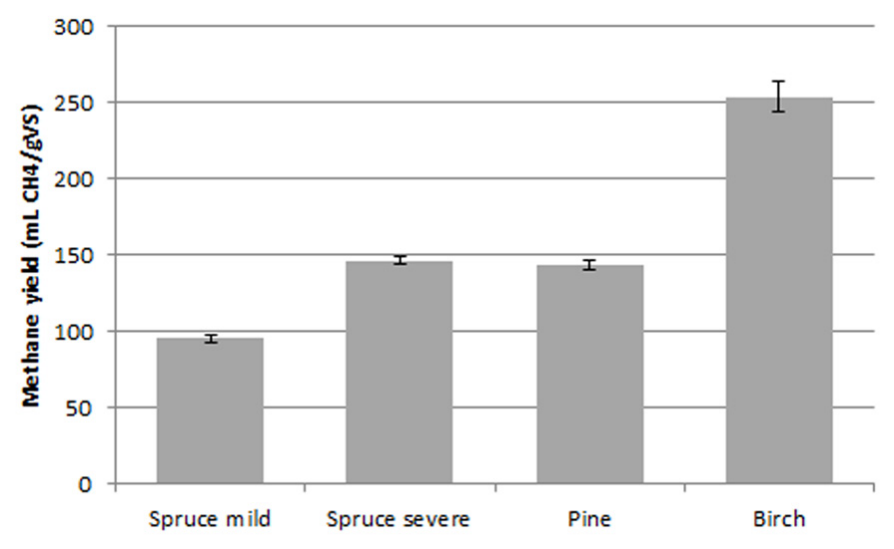
**Methane yield from the hydrothermally pretreated materials**.

**FIGURE 4:**
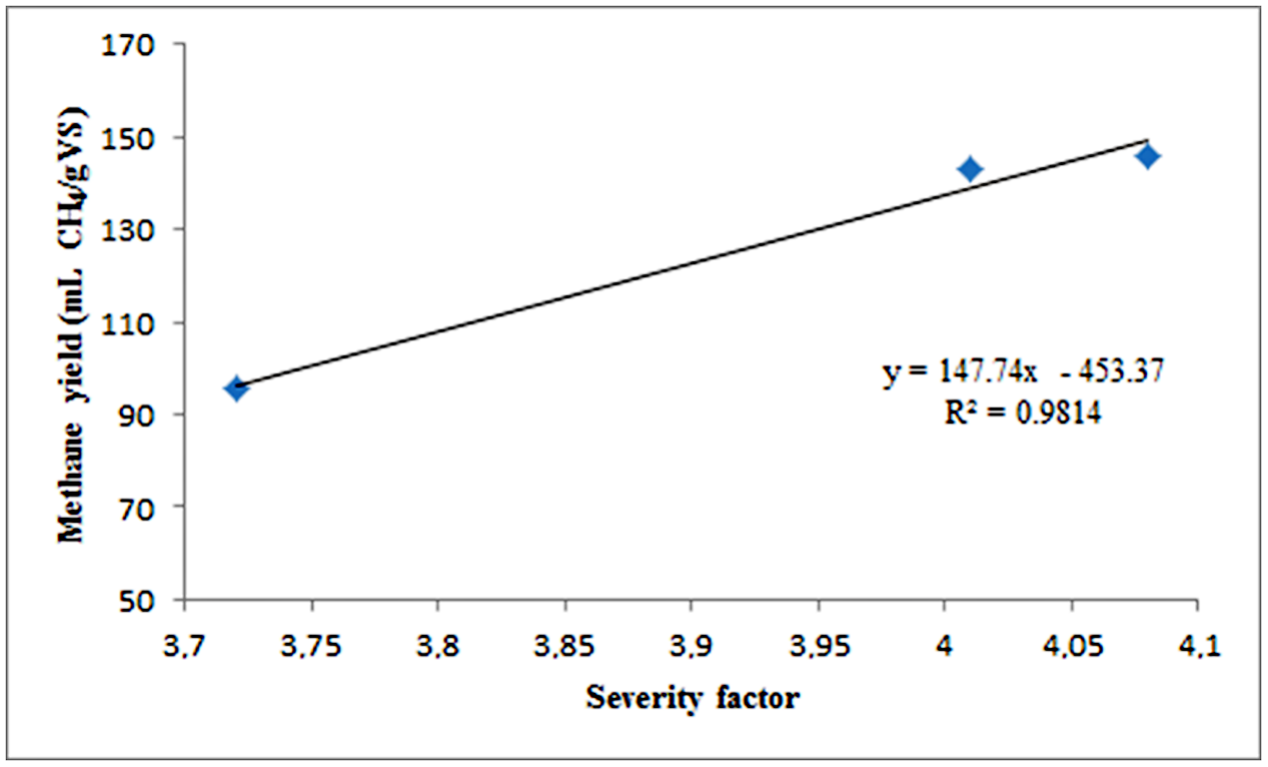
**Correlation between methane yield and SF of the pretreatment**.

In the next step, the effect of the pretreatment on the anaerobic sludge was also evaluated. As can be seen in **Figure 3**, the methane yield was greatly improved from all types of forest materials relative to that without pretreatment. The highest methane yield was obtained with birch, reaching 254.1 ± 3 mL CH4/g VS, which was approximately 14.5 times higher than the yield obtained from untreated birch. On the other hand, the lowest yield was observed with mildly pretreated spruce, reaching only 95.4 ± 2.5 mL CH4/g VS. When softwood materials were used, there was a linear increase in methane yield as SF increased (**Figure 4**).

### Effect of Detoxification of the Slurries on Methane Yield

During this work, we studied the effects of enzymatic and chemical approaches (based on the use of laccase and sodium dithionate, respectively) for detoxification of the inhibitors that were generated. The enzymatic treatment took place for 12 h prior to anaerobic digestion in order to let the enzyme act in the presence of oxygen. The enzyme load was set at 10 U/g VS. On the other hand, the chemical treatment took place simultaneously with the digestion.

**FIGURE 5:**
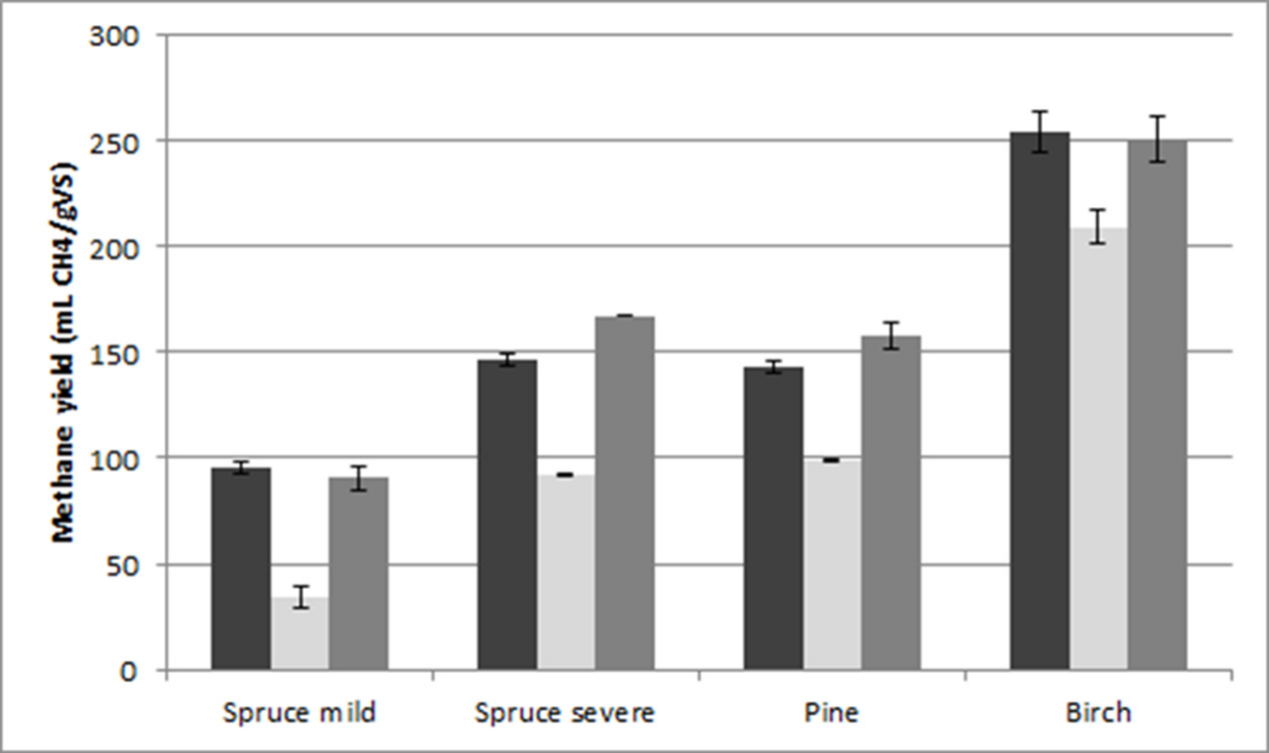
**Methane yields from undetoxified material (first bar), material detoxified with sodium dithionite (second bar), and material detoxified with laccase (third bar)**.

When 10 mM sodium dithionite was used, a concentration that was found previously to be optimal for detoxification of spruce hydrolysates for ethanol production (Alriksson et al., 2011), it was inhibitory to the anaerobic digestion (data not shown), and this inhibitory effect was still observed to some extent even when the concentration was reduced to 1 mM (**Figure 5**). These results indicate that despite the fact that sodium dithionite can be used to detoxify hydrolysates for ethanol fermentation, it is not appropriate for use in anaerobic digestion. On the other hand, when laccase was used for detoxification, it proved to be more effective for forestry-derived feedstocks that had been pretreated under more severe conditions and improved the methane yields from spruce and pine by 14.3 and 9.8%, respectively (**Figure 5**).

### Effect of Cellulase Treatment on the Methane Yield

**FIGURE 6:**
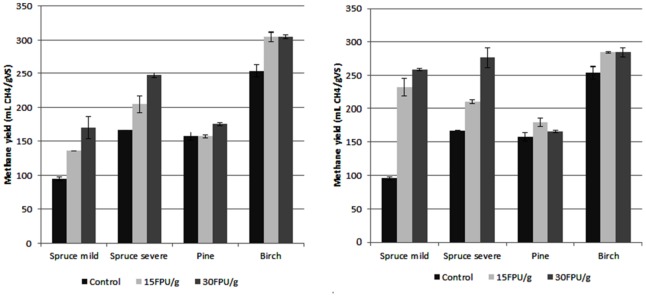
**Effect of enzymatic treatment on the methane yields, when it took place prior to **(A)** and along with **(B)** the digestion.** The first bar represents the control without addition of enzyme, and the second and third bars the addition of enzyme at 15 FPU/g and 30 FPU/g load, respectively.

Two different process configurations were examined: hydrolysis at the same time as anaerobic digestion and a separate saccharification step before digestion. In both configurations, two enzyme loadings (15 and 30 FPU/g VS) were used. Addition of enzymes improved the methane yields in both process configurations, with both enzyme loads (**Figure 6**). Although both processes were beneficial, the pre-saccharification treatment gave higher methane yield than simultaneous treatment for all the materials except birch. Of all the materials, pine showed the lowest degree of improvement when enzymes were included. On the other hand, the highest improvement was observed with spruce (both mildly and severely pretreated). The highest methane yield was obtained from birch when enzymatic treatment was performed simultaneously with the digestion, using 15 FPU/g VS enzyme load. This yield reached 304.8 ± 6.35 mLCH_4_/g VS, whereas increasing the enzyme load did not improve the methane yield further.

## Discussion

During this work, different source of forest biomass was evaluated as raw material of anaerobic digestion. Initially, the ability of digesting the forest materials without any sort of treatment was evaluated. Despite the fact that high methane yields from untreated agricultural residues have been demonstrated (Bauer et al., 2009; Lei et al., 2010), forest residues are considered to be ‘tougher’ raw materials, and for this reason the yields obtained from untreated materials are often very low (Nakamura and Mtui, 2003; Yoshida et al., 2010). Similar (low) results for methane yield were also obtained during this work when untreated materials were used reaching a highest value of 17.5 ± 1.9 mL CH_4_/g VS when birch was used (**Figure 1**). The addition of cellulolytic enzymes facilitated the anaerobic digestion and improved the methane yields obtained from the forest materials. From the results described above, it can be concluded that untreated forest materials cannot be used as raw materials for anaerobic digestion, as the yields obtained were very low even with the addition of cellulase.

Generally, when lignocellulosic materials are used for anaerobic digestion, it is believed that the rate-limiting step of the process is the hydrolysis of the polysaccharides (holocellulose or cellulose and hemicellulose) to monomeric sugars (Parawira, 2012). Due to the complex nature of holocellulose and lignin, untreated materials are highly resistant to microbial attacks. In order to reduce the resistance of holocellulose to degradation, some kind of pretreatment is necessary prior to digestion (Bruni et al., 2010). During our work a hydrothermal pretreatment was applied, where sulfur dioxide was used as catalyst. Hydrothermal pretreatment is considered as effective method to treat lignocellulosic biomass and has extensively studied during ethanol production from lignocellulosic biomass (Negro et al., 2003; Matsakas and Christakopoulos, 2013; Nitsos et al., 2013; Paschos et al., 2015). All the pretreated materials demonstrated an increase in the methane yield reaching a highest value of 254.1 ± 3 mL CH_4_/g VS when birch was used. The yields obtained from pine and spruce were lower compared to the birch, with the mild pretreated spruce resulting in the lowest results of all the pretreated materials. The difference in the methane yields obtained when birch was used and when the other forest materials were used can be attributed to the fact that birch is a hardwood, whereas spruce and pine are softwoods. Softwoods are known to be more recalcitrant than hardwoods (Kim and Hong, 2001; Palonen et al., 2004; Mirahmadi et al., 2010), and this difference in resistance is commonly attributed to the different types of lignin, as hardwoods contain a mixture of guaiacyl and syringyl units and softwoods have mainly guaiacyl units in lignin (Taherzadeh and Karimi, 2008). This could explain the lower yields achieved with softwoods during this work.

The same great improvement in methane yields was also described by Salehian and Karimi (2013), from pine branches when they were pretreated with alkali. Same positive effect of pretreatment on the methane yield from lignocellulosic materials was also found with birch after steam explosion (Vivekanand et al., 2013), with spruce and pine after alkaline pretreatment (Mirahmadi et al., 2010), and with spruce pretreated with ionic liquids (Teghammar et al., 2012).

Although pretreatment process results in an easier hydrolysable biomass and subsequent higher methane formation, inhibitory compounds can be formed during this process. These compounds are mainly derived either from the carbohydrate fraction or the lignin fraction. These inhibitors can be hydroxymethylfurfural (HMF) from the degradation of C6 sugars, furfural from the degradation of C5 sugars, and polymeric lignin or aromatic compounds—such as syringaldehyde or 4-hydroxybenzoic acid—from the degradation of lignin. Moreover, weak organic acids can be produced, such as formic acid and acetic acid. During anaerobic digestion, the aliphatic acids are not a problem (in contrast to ethanol fermentation), as they can be consumed during the methanogenesis stage. Moreover, HMF and furfural were not found to be inhibitory during digestion of xylose, and they could be consumed by the sludge and produce methane (Barakat et al., 2012). On the other hand, phenolic compounds that are released from lignin, such as phenol, cinnamaldehyde, or 4-hydroxybenzoic acid, can be potential inhibitors of the anaerobic sludge (Sierra-Alvarez and Lettinga, 1991; Hernandez and Edyvean, 2008). Such phenolic compounds have been detected in the hydrolysates of wet oxidized and steam exploded sugarcane bagasse (Martín et al., 2007; Kayembe et al., 2013).

Enzymatic treatments based on laccase have been used to improve the fermentability of pretreated slurries by reducing the amount of phenolic compounds (Jönsson et al., 1998; Jurado et al., 2009; Moreno et al., 2012). On the other hand, the benefit of using chemical reducing agents for detoxification is that they can be applied during the fermentation, and there is no need for a separate step (Cavka et al., 2011). It was also mentioned that generally sulfur oxyanions (like sodium dithionite) act by sulfonate aromatic compounds and furan aldehydes (Cavka et al., 2011). Sodium dithionite has proven to be beneficial for improvement of the fermentability of pretreated lignocellulosic biomass (Alriksson et al., 2011; Xiros and Olsson, 2014). Despite the fact that treatment with sodium dithionite was effective in reducing the inhibitory effect during ethanol fermentation, during our trial it resulted in decrease in methane yields (**Figure 5**). On the other hand, laccase treatment improved the methane yields on severe treated spruce and pine. In both materials, the SF was higher than 4 and this could have resulted in the generation of more inhibitors than with the materials that were treated at lower SF. For the materials that were pretreated at lower SF, laccase treatment did not have any effect on the methane yields. The difference in the effect of laccase detoxification can also be explained by the concentration of inhibitors in the liquid fraction. As can be seen from **Table 2**, the amounts of HMF and furfural were higher in the severely pretreated spruce and pine, following by birch and mildly pretreated spruce. Moreover, levulinic acid was only detected in severely pretreated spruce and pine (**Table 2**).

Finally, we examined the effect of cellulase treatment of the slurries on methane yields. As mentioned previously, the hydrolysis of carbohydrates to monomeric sugars is considered to be the rate-limiting part of the anaerobic digestion of lignocellulosic biomass. The consortium present in the anaerobic sludge has the ability to secrete lignocellulolytic enzymes and break down polymeric carbohydrates. On the other hand, it has been shown that the addition of external enzyme solution can increase the efficiency of the decomposition of insoluble carbohydrates (Antonopoulou and Lyberatos, 2013; Matsakas et al., 2014). The use of cellulolytic enzymes to enhance the methane yield when lignocellulosic biomass is used as raw material is not very common practice in the literature, despite the positive effect that it has. On the other hand, enzymes are used more on other materials such as wheat grains (Sonakya et al., 2001), household solid waste (Rintala and Ahring, 1994), and dairy industrial wastewater (Mendez et al., 2006). It was also demonstrated that the stage at which enzymes are added has an important effect on the methane yield. For example, Romano et al. (2009) concluded that addition of enzyme—either at the same stage of digestion or as a pre-hydrolysis—did not improve the methane yield from Jose Tall wheat grass. On the other hand, they found an improvement from 220 mLCH_4_/g VS to 290 mLCH_4_/g VS when the enzymes where added at the first stage of a two-stage anaerobic digestion. During our work, presence of enzymes was beneficial for the methane yield independently the step that the enzymes were included (either prior or simultaneous to digestion) and enzyme load. Generally, the simultaneous enzymatic treatment was more efficient for all the materials except birch. Addition of higher enzyme activities was beneficial for spruce and prehydrolyzed pine. On the other hand, higher enzyme activities did not affect the methane yield from birch and had a negative impact on pine when was treated simultaneously with the digestion. The highest results were obtained with birch, reaching 304.8 ± 6.35 mLCH_4_/g VS when an enzyme load of 15 FPU/g VS was applied prior to digestion. This is in good agreement with the results obtained before, where birch was found to be the most efficient raw material irrespective of the treatment. Forest materials have been also used by other researchers after applied different kind of pretreatments (**Table 3**). Birch is proven to be the most efficient material to be used for anaerobic digestion, whereas the application of pretreatment always resulted in an increase in the methane yields. Although some works demonstrated higher methane yield, the improvement of the methane yield compared to the untreated materials achieved during our work was the highest (**Table 3**).

**Table 3:** Comparison of the results obtained during this work with other research works in the literature.

Material	Pretreatment	Type of digestion	Yield of untreated	Yield of treated	% improvement	Reference
			(mL CH_4_/gVS)			
Spruce	Alkali	Thermophilic	30	50	74	Mirahmadi et al., 2010
Birch	Alkali	Thermophilic	250	460	83	Mirahmadi et al., 2010
Spruce	Ionic liquids	Thermophilic	66	245	271	Teghammar et al., 2012
Spruce	NaOH/thiourea	Thermophilic	30	210	600	Mohsenzadeh et al., 2012
Birch	NaOH/thiourea	Thermophilic	230	360	57	Mohsenzadeh et al., 2012
Pine	Alkali	Mesophilic	65	178	181	Salehian et al., 2013
Mixture of spruce and pine	Ionic liquids	Thermophilic	70	150	114	Kabir et al., 2014
Mixture of spruce and pine	Organosolv	Thermophilic	50	340	580	Kabir et al., 2015
Spruce	Hydrothermal	Thermophilic	10	276	2660	Present work
Pine	Hydrothermal	Thermophilic	5	180	3500	Present work
Birch	Hydrothermal	Thermophilic	18	305	1594	Present work

## Conclusion

We have demonstrated that forest residues can be efficient raw materials for anaerobic digestion. Due to their complex structure, pretreatment of the materials is necessary in order to increase the methane yield. During our work, we found that the hydrothermal pretreatment can rapidly increase the methane production from all the materials used. When the pretreatment takes place under severe conditions, a detoxification step is necessary. We also found that detoxification with sodium dithionite resulted in partial inhibition of the process—in contrast to ethanol fermentation, where this detoxification is beneficial. On the other hand, detoxification with laccase can improve the methane yield. Finally, incorporation of an enzymatic treatment results in a further improvement in the methane yields. Treatment performed along with the digestion was found to be beneficial for spruce and pine, whereas pre-saccharification was more appropriate for birch. All the materials were successfully used for the efficient anaerobic digestion resulting in the high yields of 179.9 mL CH_4_/g VS and 304.8 mL CH_4_/g VS for pine and birch, respectively, and 259.4 mL CH_4_/g VS and 276.3 mL CH_4_/g VS for mildly and severely pretreated spruce, respectively. These yields are among the highest demonstrated in the literature, whereas we also demonstrated the ability of facilitating the anaerobic digestion by enzymatic detoxification and saccharification.

## Author Contributions

All authors (LM, UR, and PC) contributed jointly to all aspects of the work reported in the manuscript. All authors have read and approved the final manuscript.

## Conflict of Interest Statement

The authors declare that the research was conducted in the absence of any commercial or financial relationships that could be construed as a potential conflict of interest.

## Footnotes

1 http://www.svenskttra.se/MediaBinaryLoader.axd?MediaArchive_FileID=eb34da70-248d-4e78-b7cf-9592aca6c0db&FileName=Forest.pdf
